# Bioactive Properties, Volatile Compounds, and Sensory Profile of Sauerkraut Are Dependent on Cultivar Choice and Storage Conditions

**DOI:** 10.3390/foods11091218

**Published:** 2022-04-22

**Authors:** Nikola Major, Iva Bažon, Nina Išić, Tvrtko Karlo Kovačević, Dean Ban, Sanja Radeka, Smiljana Goreta Ban

**Affiliations:** 1 Institute of Agriculture and Tourism, Department of Agriculture and Nutrition, K. Hugues 8, 52440 Poreč, Croatia; nikola@iptpo.hr (N.M.); iva@iptpo.hr (I.B.); nina@iptpo.hr (N.I.); tvrtko@iptpo.hr (T.K.K.); dean@iptpo.hr (D.B.); sanja@iptpo.hr (S.R.); 2 Centre of Excellence for Biodiversity and Molecular Plant Breeding, Svetošimunska 1, 10000 Zagreb, Croatia

**Keywords:** fermentation, cabbage, phytochemical composition, traditional cultivars, cold storage, sensory analysis, bioactive compounds

## Abstract

Sauerkraut is produced by cabbage fermentation either spontaneously or by adding lactic acid bacteria. Although commercial cabbage cultivars are more desirable due to their higher yield and uniformity, traditional cultivars are highly prized for their unique sensory characteristics and suitability for fermentation. The aim of this study was to investigate the properties of sauerkrauts from traditional cabbage cultivars (‘Brgujski’ and ‘Žminjski’) compared to commercial samples, and to unravel the effects of ambient (18 °C) and cold storage (4 °C) on sauerkraut properties. Higher total phenolic contents and total antioxidant capacities measured by both FRAP and DPPH methods were observed for sauerkrauts from traditional cultivars. In total, 32 volatile compounds were identified, and differences in the volatile profile were observed among the investigated sauerkrauts. The sensory properties of traditional cabbage cultivars were on par, or even better, compared to those of commercially available sauerkraut products. The cold storage conditions characteristic of commercial environments preserved the total antioxidant capacity, the red to green color ratio (*a*), as well as the lightness (*L*) of sauerkraut compared to the ambient temperatures characteristic of domestic conditions, indicating the preservation of bioactive compounds responsible for the purple cabbage head coloration of the investigated traditional cultivars.

## 1. Introduction

Cabbage (*Brassica oleracea* L. var. *capitata*) is a widely cultivated cruciferous vegetable belonging to the *Brassicaceae* family. The leaves are formed into characteristic heads that vary in shape and color, resulting in a great number of cabbage cultivars [1]. Cabbage is among the most important dietary vegetables in part due to its wide availability, relatively low production cost, and because it can be consumed in various culinary forms (fresh, fermented, cooked, etc.) [2]. According to the FAO (Food and Agriculture Organization of the United Nations), more than 70 million tons of cabbage (alongside other Brassicas, excluding cauliflower and broccoli) were produced in 2020 [3].

Recently, together with rapidly growing science-based evidence, the focus of the consumers has been shifted to a healthier diet and the consumption of food that positively influence human health [4]. Cabbage consumption is a notable source of phytonutrients in the human diet [4], and a variation in some phytochemicals has been observed in different cabbage genotypes [5,6]. Traditional landraces and local cabbage cultivars are found around the world, as well as in Croatia, representing an important source of genetic diversity [7]. Although commercial cabbage cultivars are more desirable from an economic standpoint due to their higher yield and uniform quality, traditional cultivars are still highly prized for their unique sensory characteristics and suitability for fermentation [8]. Therefore, it is crucial to recognize and preserve traditional cabbage landraces.

The majority of cabbage is consumed fresh, but sauerkraut is also a staple food item in many parts of the world [9]. Sauerkraut is produced by the spontaneous fermentation of shredded and salted cabbage leaves or by the addition of lactic acid bacteria (LAB). Traditionally, it is sliced into thin strips and spontaneously fermented by the autochthonous LAB present on the raw substrate [4,10]. As such, it is an ample ingredient in Eastern and Central European cuisine [11].

Fermentation is a valuable biotechnological process that can maintain and improve the safety, nutritional, sensory, and shelf-life properties of vegetables. The preservative effect of LAB fermentation can be attributed to the production of organic acids (lactic and acetic) that lower the substrate pH [10]. Moreover, the bioavailability of nutrients contained in raw cabbage increases with fermentation. In addition, LAB have the ability to produce volatile compounds that influence the aroma profile of the fermented cabbage [11].

The antioxidant capacity of vegetables strongly depends on genetic and environmental conditions, but also on the food processing and storage conditions [12]. According to Kusznierewicz et al. [13], the fermentation process greatly improves the antioxidant properties of cabbage. Cabbage varieties with red coloration possess higher antioxidant capacity compared to green varieties due to their increased total phenolic content, total flavonoid content, and especially anthocyanin content [14]. During the fermentation process, the total phenolic and total anthocyanin contents decrease, but, at the same time, the antioxidant capacity of the fermented cabbage increases due to LAB, which can produce a broad spectrum of compounds responsible for the beneficial properties of fermented cabbage [10].

Often, the result of fermentation is a product with specific organoleptic attributes [15]. Sauerkraut is often eaten as an adjuvant with other foods to make them more digestible, but also to increase flavor, meaning that it is highly appreciated for its particular sensory characteristics [16].

The objective of this research was to evaluate the impact of ambient and cold sauerkraut storage conditions of two local cultivars of cabbage, cv. Brgujski and landrace Žminjski, on their physicochemical characteristics and sensory quality, as well as to compare them with three commercially produced sauerkrauts available on the local market.

## 2. Materials and Methods

### 2.1. Plant Material, Sauerkraut Production, and Sampling

Cabbage cv. Brgujski is listed on the List of Varieties of the Republic of Croatia as a protected variety, whereas landrace Žminjski is maintained as a part of the gen-bank collection of the Institute of Agriculture and Tourism in Poreč (Croatia). Both cultivars are commonly grown by local farmers and used as fresh or processed cabbage. The morphological characteristics and agronomic traits of ‘Brgujski’ were described by Bažon et al. [7], while ‘Žminjski’ is considered a white cabbage variety, although purple head coloration is frequently observed (unpublished results).

Transplants of ‘Brgujski’ and ‘Žminjski’ cabbages were planted with a distance of 0.7 m between rows and 0.5 m in the row at 3–4 leaf stages in July 2020 at the Institute of Agriculture and Tourism in Poreč’s (Croatia) experimental field. Standard growing practices for cabbage were applied according to Lešić et al. [1].

Only solid, healthy, and undamaged cabbage heads were harvested at the end of October, at the stage of technological maturity. The outer leaves and inner stem were removed. The cabbage was manually shredded into approximately 3 mm shavings with the use of a slicer and placed into 70 L sterile plastic tanks. Sodium chloride (2.5% *w/w*) was evenly mixed into the shredded cabbage and a weight was applied on top to keep the cabbage under the brine. The cabbage was left to spontaneously ferment in a dark cellar at 18 °C [17], and the fermentation process was performed in triplicate for each cultivar.

After 37 days, the sauerkraut was divided into two batches for each tank, where one batch was closed in a vacuum sealer bag and stored at 4 °C [18] to represent the cold storage condition characteristic of commercial conditions, while the second batch was further stored in the tank at 18 °C to represent the ambient storage setting characteristic of domestic conditions. After 52 days, the samples were taken for laboratory analysis and sensory evaluation. For comparison with the traditional cultivars, three available commercial sauerkraut products (CS-1, CS-2, and CS-3) were purchased on the local market. The samples were immediately transferred to the laboratory. Prior to laboratory analyses, the samples were homogenized by a hand blender. The extracts for total antioxidant capacity and total phenolic content determination were obtained by ultrasound-assisted extraction (MRC DC-250, Holon, Israel) of the homogenized sauerkraut samples (250 mg) in 1.5 mL of aqueous methanol (methanol/water, 80/20, *v/v*) for 30 min. Afterward, the samples were left to macerate on an orbital shaker for 3.5 h and subsequently centrifuged at 16,000× *g*. The supernatants were transferred to a clean tube and stored at −80 °C until further analysis.

### 2.2. Physicochemical Measurements

#### 2.2.1. pH and EC Measurements

The fermentation process was monitored by measuring the brine pH and electrical conductivity (EC) in each tank. The pH results were obtained using a Seven2Go S2-Basic portable pH meter (Mettler Toledo, Columbus, OH, USA) according to ISO10523:2008, and EC was measured with a FiveGo F3 portable conductivity meter (Mettler Toledo, Columbus, OH, USA) according to ISO7888:1985. After the completion of the storage period for both cold and traditional storage, pH and EC measurements were taken from the homogenized samples.

#### 2.2.2. Dry Matter Determination

Dry matter was determined by drying the sauerkraut samples at 105 °C until constant weight in a forced hot air-circulation oven (Memmert UF160, Schwabach, Germany) according to ISO11465:1993.

#### 2.2.3. Color Evaluation

The color of the samples was determined by measuring the lightness (*L*), ratio of red and green color (*a*), and ratio of yellow and blue color (*b*) with a MiniScan EZ 4500 Portable Spectrophotometer (HunterLab, Reston, VA, USA) according to Jaiswal et al. [19].

### 2.3. Phytochemical measurements

#### 2.3.1. Total Antioxidant Capacity

The total antioxidant capacity of sauerkraut extracts was determined by the FRAP assay [20] and the DPPH radical scavenging activity assay [21]. Briefly, 100 µL of the sample extract was mixed with 200 µL of either freshly prepared FRAP reagent or 0.02 M DPPH radical for the FRAP or DPPH assays, respectively. The antioxidant capacity using the FRAP assay was determined after 10 min of reaction at 25 °C by reading the absorbance at 593 nm (Tecan Infinite 200 Pro M Nano+, Männedorf, Switzerland), while the DPPH radical scavenging capacity was determined after 30 min of reaction at 25 °C by reading the absorbance at 517 nm (Tecan Infinite 200 Pro M Nano+, Männedorf, Switzerland).

The FRAP values were calculated against a Trolox^+^ calibration curve (*y* = 0.0168*x* − 0.002; serial dilutions of Trolox—2, 5, 10, 25, 50, 75, 100 µM; coefficient of determination, *R*^2^ = 0.9999, recovery: 101.8 ± 1.6%) and expressed as nmol TE/100 g FW. The DPPH radical scavenging ability values were calculated against a standard curve of Trolox (*y* = −0.0137*x* + 0.0133; serial dilutions of Trolox—2, 5, 10, 25, 50, 75, and 100 µM; coefficient of determination, *R*^2^ = 0.9997, recovery: 103.7 ± 1.2%) and expressed as nmol TE/100 g FW, respectively.

#### 2.3.2. Total Phenolic Content

The total phenolic content (TPC) was determined using the Folin–Ciocalteu assay [22]. Briefly, 20 µL of the sample extract was mixed with 140 µL of 0.2 M Folin–Ciocalteu reagent and, after one minute, 140 µL of 6% sodium carbonate was added. The reaction mixture was incubated at 25 °C for 60 min and the absorbance was read at 750 nm (Tecan Infinite 200 Pro M Nano+, Männedorf, Switzerland). TPC was standardized against gallic acid and expressed as mg of gallic acid equivalents per 100 g sample in FW. The results were calculated against a standard curve of gallic acid (*y* = 3.7867*x* − 0.2144; serial dilutions of gallic acid—12.5, 25, 50, 75, 100, 150, and 250 mg/L; coefficient of determination, *R*^2^ = 0.9999, recovery: 102.0 ± 2.9%) and expressed as mg GAE/100 g DW.

#### 2.3.3. Volatile Compounds Analysis

The volatile compounds analysis was performed by headspace-gas chromatography/mass spectrometry (HS-GC/MS). Briefly, 2 g of the homogenized sauerkraut sample was weighed in a 20 mL headspace vial, mixed with 6 mL of deionized water spiked with the internal standard, and immediately capped. The internal standards used were 2-octanol and methyl-nonanoate. Headspace extraction was performed on an autosampler equipped with a heated agitator and a 2.5 mL heated headspace syringe (AOC6000, Shimadzu, Kyoto, Japan) using the following parameters: agitator temperature 40 °C; incubation time 45 min; headspace syringe temperature 80 °C; and volume of the sampled headspace 1 mL. The separation of the volatile compounds was performed on a Rxi 5-MS column (Restek, Bellefonta, PA, USA) by the splitless injection of 1 mL of the sampled headspace with a helium column flow of 1 mL/min and with a temperature program of: hold 40 °C, 5 min; ramp to 220 °C, 10 °C/min; ramp to 300 °C, 15 °C/min; hold 300 °C, 5 min (GC2030, Shimadzu, Kyoto, Japan). The following MS parameters (TQ8040NX, Shimadzu, Kyoto, Japan) were used: ion source temperature of 280 °C; interface temperature of 300 °C; electron impact ionization; and mass scan range from 40 to 350 m/z. For each compound, the Kovat’s retention index was calculated against a mix of standard alkanes using the same temperature program. Compounds were identified using the NIST17 database. The obtained areas under the peak were normalized against the internal standard [23].

### 2.4. Sensory Analysis

Sauerkraut sensory evaluation was performed by 10 trained panelists in a room designed according to the ISO 8589 standard, free of interference in terms of noise, visual stimulation, and ambient odor. Samples were randomly numbered and kept in plates at room temperature prior to tasting, and covered with aluminum foil [24]. Water and bread were provided to the panelists between the samples. For quantitative descriptive analysis (QDA), a 10-point scale was used to rate the intensity of attributes, where 0 represented the lowest intensity, whereas 10 represented the highest intensity [24]. The attributes of sauerkraut that the sensory panel rated were white color, red color, fresh cabbage odor, sulfur odor, off-odor, hardness, crunchiness, juiciness, fresh cabbage taste, sulfur taste, sweetness, saltiness, sourness, bitterness, astringency, off-taste, and overall quality according to Johannigsmeier et al. [24]. A hedonistic scale with 7 points was developed to rate the attributes of sauerkraut by the same panel (like strongly, like moderately, like slightly, neither like nor dislike, dislike slightly, dislike moderately, and dislike strongly) [25]. The attributes rated on the Hedonistic scale were color, sulfur odor, off-odor, hardness, crunchiness, juiciness, fresh cabbage taste, sulfur taste, sweetness, saltiness, sourness, bitterness, astringency, off-taste, and overall quality according to Johanningsmeier et al. [24] and Martinez-Villaluenga et al. [26]. The description of each sensory attribute is presented in Supplementary Table S1.

### 2.5. Statistical Analysis

The obtained physicochemical and phytochemical results were processed by Analysis of Variance (ANOVA), and homogenous groups were tested by Tukey’s post hoc test. A multivariate approach using Partial Least Squares-Discriminant Analysis (PLS-DA) was employed for data comparison and interpretation. The sensory scores were analyzed by a two-way mixed model, with the samples as the fixed effect and the sensory panel as the random effect. All statistical analyses were performed in Statistica 13.4 (Tibco Inc., Palo Alto, CA, USA).

## 3. Results and Discussion

### 3.1. Changes in Brine pH and EC during Fermentation

**Figure 1:**
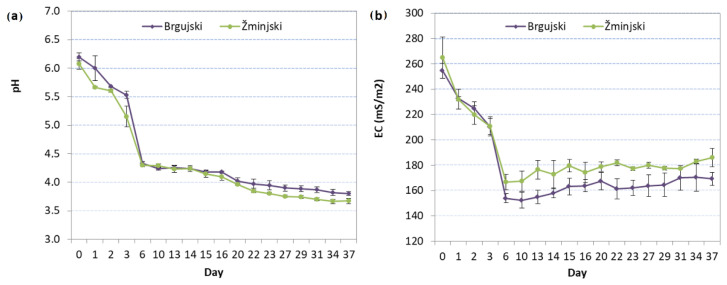
Changes in (**a**) pH and (**b**) EC (mS/m) of cabbage ‘Brgujski’ and ‘Žminjski’ during fermentation.

Parameters such as pH and EC are regularly monitored to evaluate the process of sauerkraut fermentation and are an excellent indication of when the process is completed [27]. The changes in pH and EC were observed during the first 37 days after setting up the experiment (Figure 1). The average pH of brine was higher for ‘Brgujski’ compared to ‘Žminjski’, and it decreased as the fermentation process continued (Figure 1). In the first six days, the pH significantly decreased for both cultivars from 6.14 (day 0) to 4.31 (day 6), and, thereafter, it decreased slowly to 3.74 on day 37 (Figure 1). According to Palani et al. [27], a rapid decrease in pH at the beginning of fermentation is of great importance for the quality of the final product as it minimizes the influence of spoilage bacteria. The same authors concluded that sauerkraut fermentation can be divided into two phases, where, in the first phase, rapid acidification is observed due to the increasing production of lactic and other organic acids, and, in the second phase, a final pH value below 4 is reached [27].

Dobričević et al. [17] reported an average pH value of 3.52 for traditional Croatian cv. Varaždinski sauerkraut and Cvetković et al. [8] reported pH values for traditional Serbian cv. Futoški sauerkraut in the range of 3.5–3.8, which is in line with our results. The results obtained in our study for day 6 are also in line with previously published data [15,27], where the cabbage pH on the fifth day was in the range of 5.31 to 4.67. Drašković Berger et al. [15] claimed that the fermentation process can be stopped after the pH value falls below 4.1 and, as such, the sauerkraut can be considered pasteurized; however, since consumers in Europe prefer a mild acid taste, the pH values of the finished products are usually between 4.1 and 3.8.

The decrease in the pH value of brine is directly linked to the acid content increase over time, which is mostly due to LAB activity. According to Huanefi et al. [10], during the natural fermentation of red cabbage, the available LAB in the raw material need to proliferate first before the production of organic acids, which results in a reduction in the pH value. Furthermore, changes in the pH value depend on the firmness and structure of cabbage tissue [17].

The EC parameter is an indicator of the salinity level by measuring the capacity of a liquid to conduct an electrical charge [28]. The EC of sauerkraut brine was not affected by the cultivar (*p* = 0.131) or the interaction of cultivar and time (*p* = 0.511), but significant differences were found between the days of fermentation (*p* < 0.001) (Figure 1). The brine EC of both cultivars continually decreased during the first 6 days (250 mS/m to 160 mS/m) and remained stable afterward (Figure 1).

### 3.2. Comparison of Traditional Sauerkraut Cultivars with Commercial Samples

Centuries of selection have led to a wide display of cabbage varieties. Since varieties of cabbage display a great diversity in appearance and quality [29], it is crucial to evaluate and protect specific local landraces of cabbage and derived products, such as sauerkraut. The traditional use of local cabbage varieties favors quality and palatable products [30], and thus can create highly valuable products worthy of further analysis.

The dry matter content of a cabbage variety is an important factor influencing the sauerkraut fermentation process [31]. The dry matter ranged from 7.44% to 9.93% in the analyzed sauerkraut samples, where ‘Brgujski’ and CS-2 had a higher dry matter content compared to CS-1 and CS2 sauerkrauts (Table 1). On the 27th and 62nd day of the cabbage hybrid ‘Bravo’ fermentation process, Drašković-Berger et al. [15] found a dry matter content of 8.29% to 6.85% and 7.77% to 5.5%, respectively. According to Dobričević et al. [17], different values of dry matter at the end of fermentation for different cultivars are due to the structure of the cabbage tissue, which, in turn, can alter the rate of salt penetration and the subsequent separation of the liquid phase. The same authors reported a dry matter content of fermented white cabbage cultivars ranging from 7.54% to 10.47%, depending on the harvest year growing conditions.

The sample CS-2 had the highest EC among the investigated sauerkraut samples, followed by ‘Žminjski’, indicating that the salt content in the samples may also be high (Table 1). The highest pH was observed in sample CS-1, followed by ‘Žminjski’, while the lowest value was observed in sample CS-2 (Table 1). While the level of added sodium chloride may influence the dynamics of the fermentation process in terms of the pH drop delay in highly salted samples, the final pH or acidity of the sauerkraut is not affected by the quantity of added salt [24,32].

The phenolic compounds found in vegetables possess several biological properties, such as antioxidant activity, which involves a free radical chain mechanism consisting of initiation, propagation, and termination steps [33]. Cabbage is a good source of dietary fiber, phytonutrients, and phytochemicals and can be consumed year-round. Studies have shown that white cabbage is rich in polyphenol [2,6]; however, red cabbage can have six times higher phenolic content than white cabbage [10]. In our study, the total phenolic content and antioxidant capacity measured by both FRAP and DPPH radical scavenging were significantly higher in traditional cultivars compared to the obtained commercial samples (Table 1).

Many authors have claimed an increase in fermented cabbage’s antioxidant potential in comparison with fresh cabbage [12,15,34]. These findings indicate that the fermentation process could be considered as one of the best methods for preserving and increasing the antioxidant activity of cabbage. This phenomenon could be explained by the fact that, during fermentation, the composition of bioactive compounds is modified by LAB, meaning that fermentation could induce the structural breakdown of plant cells and subsequent liberation of phenolic compounds, as well as the synthesis of new compounds [10]. According to Jakobek et al. [6], the difference between cabbage cultivars regarding their antioxidant activity can be attributed to the genotype of the used cultivar. Additionally, the difference between cultivars is often multiplied under the effect of the environment. Furthermore, the season of planting and the length of the growing period also affect the phenolic compounds in cabbages [35,36]. Since, in this research, CS-1, -2, and -3 were purchased on the local market as commercially available sauerkraut products, the difference between them and their traditional counterparts (‘Brgujski’ and ‘Žminjski’) could have been caused by numerous factors, such as growing technology, different fermentation and processing methods, storage conditions, as well as previously mentioned genotype and environmental conditions [10,12,13,15,34]. However, according to Kusznierewicz et al. [13], preservation and production technologies are believed to be responsible for antioxidants depletion in foods.

Sauerkraut sample ‘Brgujski’ had the lowest (*L*) and (*b*) values and the highest red and green ratio (*a*), which is due to its purple head coloration [7] (Table 1). ‘Žminjski’ showed comparable (*b*) and significantly lower *L*-values compared to samples CS-1 and CS-3, and a higher ratio of red and green color (*a*) compared to commercial samples, which was also due to the slight purple head hue, although this cabbage landrace is considered to be white (Table 1).

Aroma, besides taste and flavor, is one of the most important characteristics of food products for consumer palatability. It is generated by the degradation of numerous compounds creating various volatile components, such as esters, alcohols, organosulfur compounds, aldehydes, ketones, and many others, which are responsible for the well-known sauerkraut aroma [37,38]. A total of 32 identified volatile compounds were found in our study, comprising 15 esters, 8 alcohols, 5 organosulfur compounds, 3 aldehydes, and 1 ketone (Table 2).

The initial stage of the fermentation, which usually lasts 24 to 48 h, results in a significant buildup of acetic acid due to LAB, which produce it, resulting in increased sourness of the product [37,39]. Additionally, the overall quantity of acetic acid could greatly differ because it depends on the metabolism of sugars and the amount of substrate available for the bacteria [37]. Volatile acetic acid makes an important contribution to the flavor and aroma of the fermented final product [39].

The analysis of variance showed comparable ethyl acetate contents between ‘Brgujski’, ‘Žminjski’, and CS-3 (Table 2). According to Satora et al. [11] and Yang et al. [38], a higher concentration of ethyl acetate can be achieved by employing certain LAB cultures. Our results showed that spontaneous fermentation can achieve comparable or even higher ethyl acetate contents compared to commercial fermentation.

Significantly higher n-propyl acetate, pentyl acetate, (E)-3-hexen-1-yl acetate, and 2-phenylethyl acetate contents were observed in traditional compared to commercial sauerkraut samples, while the opposite was determined for butyl acetate (Table 2). Furthermore, traditional sauerkraut samples were significantly higher in their contents of hexyl acetate, (Z)-3-octen-1-yl acetate, and Neryl acetate in the case of ‘Žminjski’, and ethyl hexadecanoate in the case of ‘Brgujski’ (Table 2). The highest octyl, nonyl, and decyl acetate contents were observed in sample CS-3 compared to other investigated samples, except in the case of nonyl acetate, where ‘Žminjski’ had a comparable content (Table 2). The ethyl octanoate content was found to be significantly higher in samples CS-1 and CS-2 compared to both traditional sauerkraut samples, as well as sample CS-3 (Table 2).

According to Yang et al. [38], sauerkrauts that undergo spontaneous fermentation show a significantly lower level of ethyl octanoate, probably due to the LAB used in the fermentation of commercial samples. In our study, two out of the three commercial sauerkrauts exhibited a higher ethyl octanoate content (except sample CS-3) compared to traditional samples. The commercial samples showing a higher value of ethyl octanoate was probably due to the LAB used in the fermentation of commercial samples.

The concentrations of acetic acid esters are dependent on the microbiota responsible for the fermentation process, so the differences in our results could be explained by the different LAB employed to produce sauerkrauts, as well as the choice of cultivars [11,38].

Most alcohols detected in the investigated samples were significantly higher in two commercial sauerkrauts: 1-octen-3-ol, 2-octen-1-ol, 2-undecanol in CS-1, and 1-hexanol, 2-heptenol, and 1-decanol in CS-2 (Table 2). Sample CS-3 had the highest content of 1-heptanol compared to the other samples, except for ‘Brgujski’ (Table 2). A significantly higher content of 1-pentanol was observed in the ‘Brgujski’ sauerkraut (Table 2). Many factors dictate the alcohol profile of sauerkraut, including the choice of cultivar, fatty and amino acid contents, and pH [11]. The formation of volatile compounds, such as 1-octen-3-ol and 2-octen-1-ol, is primarily affected by pH at values between 5.0 and 5.5, more so if the cabbage is cut or crushed [11]. The alcohols detected in our study were also found in previously published studies [2,11,38].

The volatile sulfur components detected in the investigated samples included dimethyl disulfide, dimethyl trisulfide, allyl isothiocyanate, 2-isothiocyanatobutane, and 4-isothiocyanatobut-1-ene (Table 2). These sulfur compounds are affected by the fermentation conditions and the formation mechanisms of various sulfide derivatives [37]. The hydrolysis of glucosinolates, which are abundant in cabbage, gives rise to isothiocyanates and many other compounds that are responsible for sauerkraut’s characteristic flavor (i.e., bitter taste) and its beneficial bioactive effects [11]. Additionally, enzymatic reactions and chemical oxidations are responsible for the changes in sulfur compounds in fermented *Brassica* vegetables during storage [40].

The sample ‘Brgujski’ had the highest level of dimethyl disulfide, while CS-1, CS-2, and CS-3 had higher levels of 4-isothiocyanatobut-1-ene, dimethyl trisulfide, and 2-isothiocyanatobutane, respectively, compared to the other samples (Table 2). Yang et al. [30] reported a significantly higher content of dimethyl disulfide in sauerkrauts that went through spontaneous fermentation with naturally occurring microbiota compared to sauerkrauts inoculated with different LAB starter cultures. Our results show that the spontaneously fermented ‘Brgujski’ had a higher dimethyl disulfide content compared to the commercial samples, but the same was not true for ‘Žminjski’ (Table 2). Since organosulfur compounds are a direct result of glucosinolate breakdown during fermentation, the initial differences in the glucosinolate content can be attributed to the cultivar used [41]. Additionally, studies conducted on kimchi, a variety of sauerkraut with Korean napa cabbage, revealed a high dimethyl disulfide content as one of the major volatile compounds generated during fermentation [37,40]. Additionally, our results are in line with the study by Müller-Maatsch et al. [42], where the authors reported that red cabbages have a higher dimethyl disulfide content compared to white cabbages.

Several studies [11,38] have shown that inoculation with LAB can lead to the production of dimethyl trisulfide, which may explain why the CS-2 and CS-3 samples contained higher levels of the compound compared to the traditional samples in our study (Table 2). Moreover, studies focused on the volatile components of commercial kimchi also reported a high content of dimethyl trisulfide [37,40]. A recent study showed how kimchi produced by spontaneous fermentation exhibits a higher 4-isothiocyanatobut-1-ene content than kimchi inoculated with LAB [38]. Our results showed that the commercial samples CS-1 and CS-2 exhibited significantly higher 4-isothiocyanatobut-1-ene contents compared to the spontaneously fermented sauerkrauts.

A lower content of allyl isothiocyanate was observed in sample CS-1 compared to that of ‘Brgujski’ and ‘Žminjski’ (Table 2). According to Peñas et al. [18], allyl isothiocyanate is one of the predominant volatile compounds in sauerkraut, whose level further increases by the hydrolysis of glucosinolates when the pH drops to around 4 during fermentation. Other authors [2,11] also detected allyl isothiocyanate in both fresh white cabbage and sauerkraut, as well as Müller-Maatsch et al. [42] in fresh red cabbage. The different results could arise from the cultivars used [2], be due to the native microbiota [38], and/or be due to the higher level of precursors that consequently lead to the production of allyl isothiocyanate [42,43].

According to Yang et al. [38], aldehydes and ketones are important contributors to aroma and flavor, with the former giving characteristic fragrances and flavors in fruits and vegetables and the latter giving distinct fruity and herbaceous aromas to fermented vegetables. Significantly lower levels of 3-octanone and 2-undecenal were observed in traditional compared to commercial sauerkraut samples (Table 2). The highest content of (Z)-2-heptenal was detected in sample CS-1, while the nonanal content was higher in sample CS-1 compared to that of the traditional sauerkrauts (Table 2). Müller-Maatsch et al. [42] and Lončarić et al. [2] reported nonanal in both red and white cabbage samples, respectively. As fermentation progresses, the level of nonanal decreases depending on the microbiota used, where inoculated sauerkrauts maintained a higher nonanal content [38]. Our results are not conclusive in this regard due to the high nonanal variability in commercial samples CS-1 and CS-2 and the lower content in sample CS-3 (Table 2).

**Figure 2:**
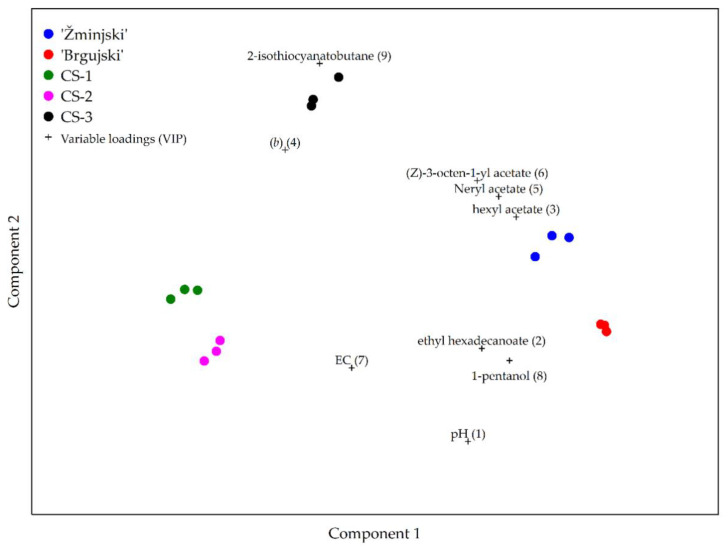
PLS-DA comparison of the investigated sauerkraut samples ‘Brgujski’ and ‘Žminjski’, and commercial sauerkrauts (CS-1, CS-2, and CS-3).

To determine which of the studied physicochemical parameters are critical in the discrimination between the traditional and commercial sauerkraut samples, partial least squares-discriminant analysis (PLS-DA) was used. The obtained model showed differences between traditional sauerkraut samples as well as commercial varieties, as seen in Figure 2.

The variable importance in projection (VIP) scores showed that the most important physicochemical parameters in the discrimination between different sauerkraut samples were pH, ethyl hexadecanoate, hexyl acetate, (*b*), Neryl acetate, (Z)-3-octen-1-yl acetate, EC, 1-pentanol, and 2-isothiocyanatobutane.

Among the investigated parameters, pH, ethyl hexadecanoate, and EC were used to differentiate between CS-1 and CS-2, as well as between the ‘Brgujski’ and ‘Žminjski’ samples (Figure 2). The differences in the EC of the samples might have been due to the different quantities of NaCl used in the production of the commercial sauerkrauts, which may range between 0.6% and 6% NaCl [10].

Since higher levels of hexyl acetate were found in the CS-3 and in the ‘Brgujski’ and ‘Žminjski’ samples compared to CS-1 and CS-2, it was used to discriminate between these groups (Figure 2). Conversely, neryl acetate and (Z)-3-octen-1-yl acetate were used to differentiate the ‘Žminjski’ sauerkraut from the other samples (Figure 2). In addition, the ratio of yellow and blue color (b) and 1-pentanol were used to differentiate ‘Brgujski’ sauerkraut from the other samples (Figure 2). Finally, a higher 2-isothiocyanatobutane content was characteristic of CS-3 and was used to differentiate it from the other samples (Figure 2).

### 3.3. Sensory Analysis of Traditional and Commercial Sauerkraut Samples

Since food is consumed not only for its nutritive value, but also for enjoyment, properties such as appearance, taste, texture, and aroma play an important role in whether a person chooses a particular food [44]. Sensory data for sauerkraut samples were obtained from a trained sensory panel by both the QDA and Hedonistic scale methods (Tables S2 and S3, respectively). Analysis of variance of the QDA data showed that the attributes white color, red color, sulfur odor, fresh cabbage odor, fresh cabbage taste, hardness, crunchiness, juiciness, saltiness, sweetness, and overall quality were significantly different between the analyzed sauerkraut samples (Table S2). After analysis of variance of the hedonistic data, the attributes sulfur odor, hardness, crunchiness, juiciness, fresh cabbage taste, sweetness, saltiness, and overall quality were found to be significantly different between the investigated sauerkraut samples (Table S3).

**Figure 3:**
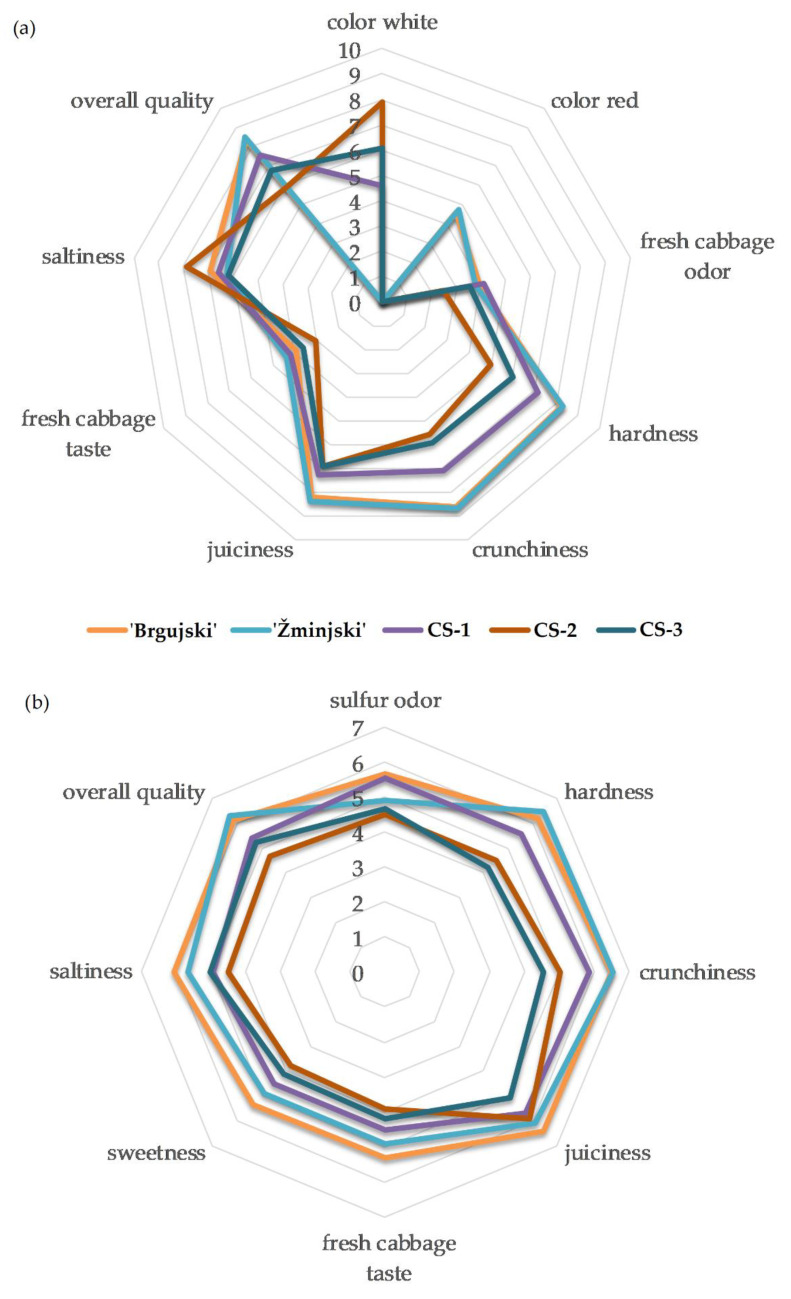
Sensory scores of sauerkraut samples from ‘Brgujski’ and ‘Žminjski’, and commercial sauerkrauts (CS-1, CS-2, and CS-3) obtained by the (**a**) QDA method and (**b**) Hedonistic scale. The results are shown only for significantly different sensory attributes after ANOVA according to Supplementary Tables S2 and S3.

A properly fermented cabbage is characterized by an appealing aroma, flavor, color, and texture [15]. The trained sensory panel distinguished commercial sauerkraut from traditional varieties primarily in terms of color, where commercial sauerkraut was described as white, while traditional samples were described as red (Figure 3a). In addition, the Hedonistic scale data had no color preference for either white or red cultivars (Figure 3b). The color perception in traditional sauerkraut by the QDA method is in accordance with our previously stated results of ‘Brgujski’ having a higher (*a*) value compared to the other samples, closely followed by ‘Žminjski’ (Table 1).

All sauerkraut samples, except CS-2, shared moderate fresh cabbage odor and taste, according to the QDA data (Figure 3a). The data obtained from the Hedonistic scale showed that the fresh cabbage taste was rated higher in ‘Brgujski’ compared to CS-1, CS-3, and CS-2 (Figure 3b). Fermented cabbage flavor is determined by components derived from the raw material, which is changed during the fermentation process, and it is a key component of sauerkraut quality. It is characterized by salty, sour, and sulfur notes [11].

Although sulfur odor was not found to be statistically different by the QDA method (Table S2), the Hedonistic scale graded this attribute higher in CS-1 and the traditional sample ‘Brgujski’ compared to CS-2 and CS-3 (Figure 3b). According to Martínez-Villaluenga et al. [26], characteristic sauerkraut sulfur notes are considered a quality attribute of properly fermented cabbage and are derived from glucosinolates. The organosulfur compound analysis obtained by HS-GCMS did not show similarities between samples CS-1 and ‘Brgujski’, where CS-1 had a significantly higher content of 4-isothiocyanatobut-1-ene, while ‘Brgujski’ had the highest dimethyl disulfide content.

The hardness, crunchiness, and juiciness attributes were consistently graded higher in the traditional sauerkraut samples compared to commercial samples by the QDA method, except for sample CS-1 for hardness (Figure 3a). The Hedonistic scale showed preference for the hardness and crunchiness of the traditional cultivars compared to commercial samples CS-2 and CS-3, while commercial sample CS-1 had comparable grades (Figure 3b). The panel also preferred the juiciness of the ‘Brgujski’ sauerkraut compared to commercial samples CS-1 and CS-3, while sauerkraut produced from ‘Žminjski’ and CS-2 had comparable juiciness to ‘Brgujski’ based on the Hedonistic scale (Figure 3b). The salt content, as indicated by the EC parameter, was higher in samples CS-2 and ‘Žminjski’, confirming that the appearance and texture of sauerkraut are also influenced by the amount of salt used in production [11].

The flavor of fermented foods is a key indicator of sensory qualities, and it often determines acceptance by consumers [45]. In addition to having the highest EC value, sample CS-2 was found to be the saltiest by the sensory panel (Figure 3a), which resulted in lower grades on the Hedonistic scale (Figure 3b). Due to rising health awareness, nowadays, customers prefer lower salt contents [10]. Additionally, excessive salt addition may contribute to a decrease in crunchiness, which is associated with an increase in osmotic pressure [11], which resulted in a low hardness grade for CS-2 with the QDA method (Figure 3a). The traditional sauerkraut ‘Brgujski’ was graded second for saltiness by the QDA (Figure 3a) and highest by the Hedonistic scale (Figure 3b). The Hedonistic scale graded the sauerkraut from traditional cultivars higher in sweetness compared to the commercial sauerkrauts CS-2 and CS-3 (Figure 3b), but the data from the QDA method showed no significant differences between the samples in this attribute (Table S2).

In terms of overall quality, the traditional sauerkraut samples were predominantly graded higher than their commercial counterparts by both the QDA (except for CS-1) and the Hedonistic scale methods (Figure 3a and Figure 3b, respectively). Our results were similar to the results found by Satora et al. [11], where the most highly rated sauerkrauts were also those rated highest in texture, with average grades for atypical qualities (off-taste). Torres et al. [46] claimed that controlled fermentation of vegetables, using selected starters, is highly recommended due to the reduced chance of fermentation failure and better sensory quality. Our results indicate that spontaneous fermentation does not negatively impact sensory characteristics in sauerkrauts.

### 3.4. Influence of Storage Conditions and Cultivar on the Physicochemical and Phytochemical Parameters of Traditional Sauerkraut Samples

Sauerkrauts can be stored in several ways, generally in cold storage (refrigerator or cellar) at low temperatures, seldom in freezing conditions [47]. After the fermentation period has run its course, the sauerkrauts are moved into cold and dry storage to preserve the nutrients, texture, and flavor [48]. Therefore, maintaining an appropriate temperature and humidity during storage is essential for preventing quality degradation [49].

Partial least squares-discriminant analysis (PLS-DA), a supervised multivariate statistical method, was used to determine which of the studied physicochemical parameters are critical in the discrimination between the ambient and cold storage of sauerkraut, as well as the difference between the two local cultivars of cabbage. The variable importance in projection (VIP) scores showed that the most important physicochemical or phytochemical parameters in descending order were FRAP (1.17), 1-decanol (1.14), ethyl octanoate (1.14), DPPH (1.11), 2-isothiocyanato butane (1.08), *L* (lightness) (1.05), 2-undecenal (1.04), (*a*) (red/green ratio) (1.02), ethyl hexadecanoate (1.01), (Z)-3-octen-1-yl acetate (1.01), and EC (1.01) (Figure 3). Significant differences using ANOVA were observed in all parameters deemed important by the obtained model and are shown in Supplementary Table S4.

For the optimal exploitation of products, such as sauerkraut, information about the changes that occur during processing and storage must be obtained [50]. FRAP had the greatest impact on the difference between ambient and cold storage, with cold storage having higher FRAP values than ambient storage (Figure 3). The antioxidant capacity of vegetables mostly depends on their genetic background and environmental conditions during growth, but can also be affected by storage conditions [12]. Furthermore, a loss of antioxidant activity may occur due to the leaching, degradation, and/or oxidation of bioactive compounds during storage [50]. Opposite to our results were the results found by Galani et al. [51], who reported a significant decrease in antioxidant activity by both the FRAP and DPPH methods when certain vegetables were exposed to cold storage at 4 °C for 15 days, although fresh cabbage exhibited the lowest loss of antioxidant activity.

The quantity of 1-decanol is also crucial in the differentiation between storage methods, as its level is found to be higher in ambient conditions compared to cold storage (Figure 3). It is well known that changes in temperature are linked to the reaction rate of almost all reactions, and the production of alcohols, such as 1-decanol, is no exception [52]. Therefore, the higher levels of 1-decanol detected in ambient storage could be due to the higher temperature setting than that in cold storage.

**Figure 4:**
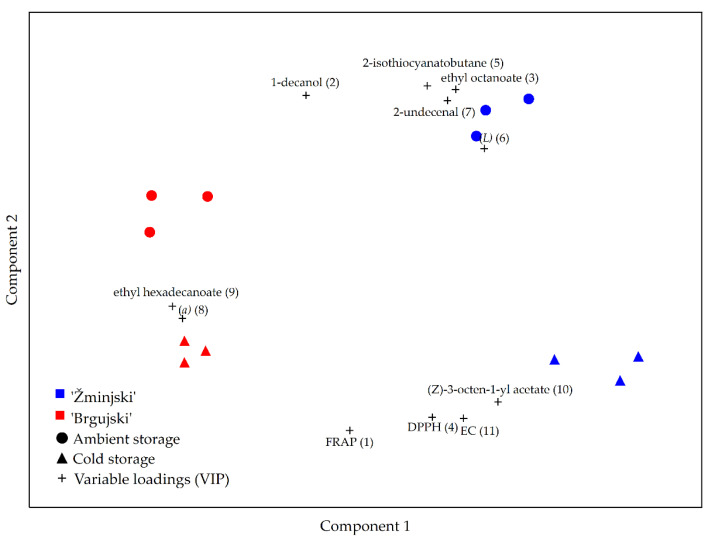
Influence of storage conditions (ambient and cold) and cabbage cultivar ‘Brgujski’ and ‘Žminjski’ on physicochemical and phytochemical sauerkraut parameters using PLS-DA.

Higher values of ethyl octanoate were characteristic of the ‘Žminjski’ and ‘Brgujski’ sauerkraut samples stored in the ambient setting compared to the values reported in samples stored in the cold setting (Figure 4). Again, the higher temperature setting of ambient storage could have influenced the production rate of ethyl octanoate, thus making this parameter highly important in the differentiation between storage conditions.

The DPPH radical-scavenging VIP score demonstrates the high influence on the difference between ambient and cold storage, with the former showing lower values compared to the latter, as reported for the FRAP values (Figure 4). According to Kapusta-Duch et al. [48], temperatures from 0 to 10 °C are optimal for the storage of most vegetables, as such temperatures limit the metabolic processes in vegetables and inhibit pathogenic microorganism growth. The identification and quantification of antioxidant activity losses can lead to storage condition optimization, consequently leading to a more desirable final product [49]. Kapusta-Duch et al. [48] also observed an increase in antioxidant activity in sauerkraut stored for 2 months in zipped low-density polyethylene bags at 4–5 °C in comparison to the samples prior to packaging.

According to Shim et al. [49], the amount of glucosinolates in Chinese cabbage varies with genetic characteristics, growth phase, climate, post-harvesting processing (storage), and several other factors, such as the plant organ, cultivation period, and cultivation conditions. Since isothiocyanates are a breakdown product of glucosinolates, their levels are consequently affected by the same factors. In addition, several other authors reported that discrepancies could have been caused due to the chemical properties (polarity and volatility) of various types of glucosinolates, overall microbiological stability, and even to the type of container used while storing the sauerkrauts [33,48]. The values of 2-isothiocyanatobutane were found to be important in discriminating between the ambient and cold storage of ‘Žminjski’ sauerkraut samples, thereby corroborating that at least several factors, such as the genotype, storage setting, type of container, chemical properties, and microbiological stability, affect the content of this compound. Meanwhile, no difference in the quantity of the 2-isothiocyanatobutane compound was observed between the storage methods of ‘Brgujski’ sauerkraut (Figure 4).

‘Brgujski’ sauerkraut samples stored in a cold setting were characterized by a lower (*L*) value compared to those in the ambient storage conditions, while no difference was observed between the storage methods of ‘Žminjski’ sauerkraut. (Figure 4). Higher (*L*) values indicate an overall lighter color, which, according to Johanningsmeier et al. [24], is a desirable quality attribute for white cabbage sauerkraut but, according to our results, the same cannot be said for cabbage with red head coloration, because higher (*L*) values indicate the loss of red pigmentation and browning of the sample. The ratio of red and green color (*a*) differs between local sauerkraut varieties, where ‘Brgujski’ has higher (*a*) values compared to ‘Žminjski’, which is due to the more intense red head coloration of ‘Brgujski’ [7], as previously mentioned. In addition, the (*a*) values were also different between storage conditions, where ‘Brgujski’ sauerkraut in the cold storage setting exhibited higher (*a*) values compared to those in the ambient storage, whereas the opposite was observed for ‘Žminjski’ (Figure 4). In the case of ‘Brgujski’, this could be due to the loss of the red color represented by anthocyanins under ambient conditions, evidenced by the loss of antioxidant activity, as shown by both the FRAP and DPPH radical scavenging assays. In the case of ‘Žminjski’, an increase in the (*a*) value under ambient conditions could be linked to the decrease in green color caused by oxidation processes and subsequent lower antioxidant activity, as shown again by both the FRAP and DPPH radical scavenging assays. Furthermore, according to Walkowiak-Tomczak and Czapski [53], the storage temperature, storage time, and pH have a significant impact on the anthocyanin content. In other words, each of the factors reduces the content of anthocyanins and, synergistically, they could exhibit a pronounced effect leading to the loss of sauerkraut coloration. Thus, changes in the anthocyanin content were reflected in color change, which was associated with the decline in color lightness/intensity and consequently evident as the overall browning of the sample.

The developed PLS-DA model showed that the quantity of 2-undecenal differed between the ‘Brgujski’ and ‘Žminjski’ sauerkraut varieties (Figure 4). According to Suriyaphan et al. [54], 2-undecenal is responsible for green and cilantro-like odor; hence, a link between its level and cabbage variety could be possible. Higher levels of 2-undecenal are characteristic of ‘Žminjski’ sauerkraut samples, especially when stored in an ambient setting. Given that the temperature has a great impact on the reaction rate of any reaction taking place, a higher content of 2-undecenal during ambient storage could be due to the higher storage temperature than that in cold storage.

In the case of the ethyl hexadecanoate content, the PLS-DA model demonstrated how it has ab impact on the differentiation between cabbage cultivars and on the differentiation between the storage settings of ‘Brgujski’, with cold storage showing higher values (Figure 4). Prior to cold storage, the cabbage samples were shredded, thus damaging the plants’ cell structure which makes the diffusion of fatty acids easier. Since particles with lower temperatures have lower kinetic energy, the diffusion rate of particles also drops. The lower temperature in cold storage affect diffusion in a way that the diffusion rate of ethyl hexadecanoate drops much more than it would in ambient storage, where the temperature is higher, explaining why the stated compound is more preserved in the colder storage setting [55].

In the samples stored in a cold setting, the (Z)-3-octen-1-yl acetate content was higher compared to that in the samples stored in an ambient setting, but only in the ‘Žminjski’ sauerkraut (Figure 4). Being one of the constituents of cabbage’s essential oil, the compound is much more diffused due to the mechanical wounding of the plant’s cell structure prior to cold storage [56]. Although it converts into its alcohol derivate by hydrolysis, the secondary alcohol formed is then converted into ketone by oxidation [57]. Both reactions are initiated due to mechanical wounding and catalyzed by enzymes [57]. The lower temperature in the cold storage significantly inhibits the enzymatic activity and, therefore, a higher (Z)-3-octen-1-yl acetate content was detected [58].

According to the developed model, EC is responsible for the differentiation between sauerkraut of local cultivars as well as between the storage conditions of ‘Žminjski’ sauerkraut. According to Żywica et al. [59], the sample mineral composition can significantly affect the EC. A possible cause as to why the ‘Žminjski’ sauerkraut sample stored in the ambient setting exhibited a lower EC value could be due to the higher storage temperature, which leads to increased diffusion of the ions and molecules into the brine, consequently decreasing the EC of the cabbage itself. However, in cold storage, due to the lower storage temperature, the diffusion of particles was slower, thus making the EC of the cabbage increase.

The sensory analysis of sauerkraut samples did not show significant differences between different storage conditions, as well as traditional cultivars (data not shown).

## 4. Conclusions

Sauerkrauts produced from traditional cultivars ‘Brgujski’ and ‘Žminjski’ showed excellent bioactive properties, with a high total phenolic content and total antioxidant capacity. The sensory scores by the QDA and Hedonistic scale method of traditional sauerkrauts produced by spontaneous fermentation were equal to or better compared to those of the commercially available sauerkraut samples. Sauerkrauts produced from traditional cabbage cultivars, especially ‘Žminjski’, were characterized by an elevated ester compound content, while commercial cultivars CS-1 and CS-2 were more abundant in alcohols. ‘Brgujski’ sauerkraut was most abundant in dimethyl disulfide, while commercial sauerkrauts CS-2 and CS-3 were more abundant in dimethyl trisulfide. Commercial sauerkrauts were also characterized by higher aldehyde and ketone contents, especially sauerkraut CS-1. The cold storage conditions characteristic of commercial environments preserved the total antioxidant capacity, the red to green color ratio (*a*), as well as the lightness (*L*) of sauerkraut compared to the ambient conditions, where higher temperatures could be observed, indicating the preservation of compounds responsible for the purple cabbage head coloration of the investigated traditional cultivars. The bioactive properties, volatile profile, and sensory scores indicate that the investigated cultivars ‘Brgujski’ and ‘Žminjski’ indeed have the potential to be a superfood. However, besides the genotype and optimal processing technology, it is necessary to have appropriate storage conditions to preserve the sauerkraut’s quality. Moreover, the obtained results open the possibility for local farmers and producers to preserve biodiversity by further exploiting traditional cultivars and creating foods with added value that can be positioned efficiently on the market and, at the same time, be interesting to a wide range of consumers.

## Figures and Tables

**Table 1:** Physicochemical and phytochemical parameters of sauerkraut samples ‘Brgujski’ and ‘Žminjski’, and commercial sauerkrauts (CS-1, CS-2, and CS-3).

	Sauerkraut Samples					
	‘Brgujski’	‘Žminjski’	CS-1	CS-2	CS-3	*p*-Value
Physicochemical parameters						
DM (%)	9.93 ± 0.02 a	9.05 ± 0.02 b	8.17 ± 0.17 c	9.7 ± 0.21 ab	7.44 ± 0.64 c	***
EC (mS/m)	110 ± 3 c	143 ± 4 b	83 ± 1 d	208 ± 15 a	96 ± 1 cd	***
pH	3.61 ± 0.01 c	3.79 ± 0.01 b	3.84 ± 0.01 a	3.58 ± 0.01 e	3.67 ± 0.01 cd	***
(*L*)	50.6 ± 1.7 d	57.0 ± 3.2 c	61.8 ± 1.9 ab	59.7 ± 1.4 bc	64.7 ± 2.8 a	***
(*a*)	23.7 ± 4.9 a	9.7 ± 0.4 b	1.7 ± 0.3 d	4.0 ± 0.2 c	1.4 ± 0.3 d	***
(*b*)	14.3 ± 1.9 c	26.6 ± 1.8 a	28.4 ± 6.6 a	19.3 ± 3.5 b	25.3 ± 1.8 a	***
Phytochemical parameters						
TPC (mg GAE/100 g FW)	34.9 ± 2.7 b	61.4 ± 4.6 a	12.7 ± 0.8 cd	14.8 ± 1.4 c	6.1 ± 0.6 d	***
FRAP (nmol TE/100 g FW)	152 ± 13 a	143 ± 5 a	52 ± 2 c	62 ± 1 c	100 ± 8 b	***
DPPH (nmol TE/100 g FW)	118 ± 6 a	122 ± 3 a	59 ± 6 d	78 ± 4 c	102 ± 1 b	***

The means (±standard deviation) with different letters indicate homogenous groups in Tukey’s post hoc test. *** *p* ≤ 0.001. (*L*)—Lightness; (*a*)—ratio of red and green; (*b*)—ratio of yellow and blue.

**Table 2:** Volatile compound contents of sauerkraut samples ‘Brgujski’ and ‘Žminjski’, and commercial sauerkrauts (CS-1, CS-2, and CS-3) (Mean ± SD, *n* = 3).

Volatile Compounds (Peak Area)	Sauerkraut Samples					
	‘Brgujski’	‘Žminjski’	CS-1	CS-2	CS-3	*p*-Value
Esters						
ethyl acetate	1,395,228 ± 295,733 a	1,078,893 ± 113,810 ab	596,995 ± 256,186 bc	45,486 ± 10,233 c	1,479,426 ± 235,640 a	***
n-propyl acetate	203,543 ± 31,519 a	111,616 ± 6090 b	54,663 ± 3490 c	16,184 ± 6650 c	25,751 ± 2671 c	***
butyl acetate	36,442 ± 36,690 c	54,892 ± 17,787 c	286,608 ± 7828 a	182,097 ± 1565 b	203571 ± 24,430 b	***
pentyl acetate	27,786,255 ± 9,993,811 a	37,351,541 ± 584,954 a	419,590 ± 24,673 b	468,125 ± 24,911 b	11,292,836 ± 2,047,968 b	***
(E)-3-hexen-1-yl acetate	1,248,049 ± 228,779 b	3,615,000 ± 539,369 a	19,495 ± 15,791 c	28,931 ± 982 c	87,931 ± 12,928 c	***
hexyl acetate	424,619 ± 64,423 b	2,256,204 ± 218,496 a	37,024 ± 6511 c	62,431 ± 21,654 c	391,433 ± 114,293 b	***
(Z)-3-octen-1-yl acetate	172,678 ± 36,116 bc	470,875 ± 73,075 a	113,805 ± 9591 c	220,182 ± 8788 bc	273,790 ± 98,313 b	***
octyl acetate	200,231 ± 31,786 b	545,995 ± 54,052 b	477,884 ± 32,921 b	208,571 ± 36,727 b	1,231,124 ± 436,888 a	***
2-phenylethyl acetate	7,123,750 ± 563,048 a	4,615,988 ± 592,564 b	171,843 ± 23,010 c	22,569 ± 331 c	290,101 ± 44,164 c	***
nonyl acetate	872,016 ± 158,370 bc	1,550,844 ± 165,832 ab	34,304 ± 12,129 c	42,746 ± 23,539 c	1,875,895 ± 703,985 a	***
Neryl acetate	37,341 ± 11,302 b	143,376 ± 31,860 a	25,183 ± 2742 b	24,130 ± 4007 b	52,416 ± 17,119 b	***
decyl acetate	38,558 ± 10,982 b	38,029 ± 5532 b	26,003 ± 1784 b	23,013 ± 4517 b	126,374 ± 41,098 a	***
ethyl octanoate	24,789 ± 6243 c	35,458 ± 395 c	117,050 ± 5893 b	223,767 ± 14,962 a	38,834 ± 5295 c	***
ethyl hexadecanoate	2,149,623 ± 96,135 a	384,260 ± 14,824 c	1,031,981 ± 65,346 b	297,926 ± 12,398 c	418,161 ± 23,793 c	***
Alcohols						
1-pentanol	2152,915 ± 264,400 a	321,111 ± 8094 bc	605,969 ± 18,756 b	115,464 ± 1806 c	40,570 ± 15,435 c	***
1-hexanol	122,337 ± 17,402 cd	162,648 ± 42,423 bc	228,462 ± 10,241 b	345,102 ± 25,981 a	85,997 ± 16,499 d	***
1-heptanol	288,268 ± 30,967 ab	215,781 ± 20,872 bc	94,463 ± 7183 d	111,842 ± 4062 cd	404,260 ± 92,168 a	***
2-heptenol	61,442 ± 26,990 b	53,295 ± 9081 b	92,208 ± 3460 b	254,405 ± 1543 a	71,346 ± 17,979 b	***
1-octen-3-ol	137,631 ± 18,890 c	180,286 ± 21,721 bc	664,844 ± 48,478 a	136,092 ± 12,520 c	303,109 ± 108,969 b	***
2-octen-1-ol	50,729 ± 3057 b	55,909 ± 11,587 b	369,649 ± 22,875 a	67,852 ± 7470 b	108,273 ± 61,388 b	***
1-decanol	19,322 ± 3443 c	15,656 ± 2510 c	41,728 ± 11,006 c	304,241 ± 5693 a	78,027 ± 20,450 b	***
2-undecanol	608,142 ± 12,494 c	490,000 ± 32,633 c	633,783 ± 79,916 a	44,555 ± 14,489 b	215,613 ± 13,970 b	***
Organosulfur compounds						
dimethyl disulfide	2,152,915 ± 264,400 a	913,856 ± 37,479 bc	638,656 ± 52,597 bc	151,616 ± 19,805 d	477,490 ± 24,467 cd	***
allyl isothiocyanate	100,499 ± 580 a	87,984 ± 45,420 a	27,750 ± 2897 b	46,326 ± 4637 ab	68,401 ± 4752 ab	**
2-isothiocyanatobutane	13,840 ± 3336 c	11,175 ± 527 c	23,557 ± 2257 b	7701 ± 2213 c	47,456 ± 4184 a	***
dimethyl trisulfide	72,585 ± 11,457 c	70,762 ± 35,987 c	92,208 ± 3460 c	254,405 ± 1543 a	176,472 ± 21,729 b	***
4-isothiocyanatobut-1-ene	117,438 ± 14,676 c	180,286 ± 21,721 c	747,624 ± 102,540 a	467,811 ± 12,336 b	294,755 ± 122,098 bc	***
Aldehydes and ketones						
3-octanone	179,080 ± 36,785 c	259,322 ± 41,905 c	903,077 ± 117,834 b	886,598 ± 88,863 b	1,805,712 ± 341,480 a	***
2-heptenal, (Z)-	85,250 ± 61,199 c	85,685 ± 44,289 c	1,576,219 ± 127,593 a	276,669 ± 40,495 b	73,943 ± 22,875 c	***
nonanal	56,889 ± 6595 b	79,719 ± 4696 b	1,906,507 ± 1,010,828 a	1,116,308 ± 970,319 ab	339,365 ± 16,105 ab	***
2-undecenal	32,541 ± 11,638 c	37,433 ± 11,719 c	2,320,481 ± 141,942 a	509,651 ± 63,846 b	319,381 ± 161,272 b	***

The means (±standard deviation) with different letters indicate homogenous groups in Tukey’s post hoc test. ** *p* ≤ 0.01, *** *p* ≤ 0.001.

## Data Availability

Data is contained within the article or supplementary material.

## Supplementary Materials

The following supporting information can be downloaded at: www.mdpi.com/article/10.3390/foods11091218/s1, Table S1: Sensory attributes used to describe sauerkraut samples; Table S2: Sensory scores of sauerkraut samples from ‘Brgujski’, ‘Žminjski’ and commercial sauerkrauts (CS-1, CS-2, and CS-3) obtained by the QDA method; Table S3: Sensory scores of sauerkraut samples from ‘Brgujski’, ‘Žminjski’ and commercial sauerkrauts (CS-1, CS-2, and CS-3) obtained on the Hedonistic scale; Table S4: ANOVA of phytochemical and physicochemical parameters with PLS-DA VIP scores equal or higher than one.
