# Supplementary material for: Bioactive Properties, Volatile Compounds, and Sensory Profile of Sauerkraut Are Dependent on Cultivar Choice and Storage Conditions
Source: Foods. 2022 Apr 22;11(9):1218. doi: 10.3390/foods11091218 (PMC9101451; doi:10.3390/foods11091218)
Supplement: Supplementary file 1 [file foods-11-01218-s001.zip › foods-1678226-supplementary.pdf]

Supplementary Table S1. Sensory attributes used to describe sauerkraut samples

Attributes	Definition (Johanningsmeier et al. 2007)	Intensity (Modified from Johanningsmeier et al. 2007)	Hedonic scale (Ritz et al., 1992; Martinez-Villaluenga 2012)
**APPEARANCE**			
White color	Graduated scale from green to creamy colour	1 = green to 10 = creamy	1 = dislike very much to 7 = like very much
Red color	Graduated scale from light purple to dark purple colour	1 = light purple to 10 = dark purple	
**ODOR PROPERTIES**			
Fresh cabbage odor	Green, vegetative aroma and flavor of raw cabbage	1 = lowest intensity to 10 = highest intensity	1 = dislike very much to 7 = like very much
Sulfur odor	The strong sulfur note that is characteristic of properly fermented sauerkraut.	1 = lowest intensity to 10 = highest intensity	1 = dislike very much to 7 = like very much
Off-odor	Open scale with space allotted for a write-in descriptor to be used when an unanticipated (and abnormal) odor is observed	Detected or not detected	Not applicable
**IMPRESSION WHILE CHEWING**			
Hardness	The amount of force or effort required for masticating the sample	1 = lowest intensity to 10 = highest intensity	1 = dislike very much to 7 = like very much
Crunchiness	Sound and feeling associated with chewing raw vegetables	1 = lowest intensity to 10 = highest intensity	1 = dislike very much to 7 = like very much
Juiciness	The amount of brine in the kraut product	1 = lowest intensity to 10 = highest intensity	1 = dislike very much to 7 = like very much
**TASTE AND RETRONASAL SMELL**			
Fresh cabbage taste	Green, vegetative aroma and flavor of raw cabbage	1 = lowest intensity to 10 = highest intensity	1 = dislike very much to 7 = like very much
Sulfur taste	The strong sulfur note that is characteristic of properly fermented sauerkraut	1 = lowest intensity to 10 = highest intensity	1 = dislike very much to 7 = like very much
Sweetness	Sweet taste. Example: sucrose in water	1 = lowest intensity to 10 = highest intensity	1 = dislike very much to 7 = like very much
Saltiness	Salty taste. Example: sodium chloride in water	1 = lowest intensity to 10 = highest intensity	1 = dislike very much to 7 = like very much
Sourness	Sour taste. Example: citric acid in water	1 = lowest intensity to 10 = highest intensity	1 = dislike very much to 7 = like very much
Bitterness	Bitter taste. Example: caffeine in water	1 = lowest intensity to 10 = highest intensity	1 = dislike very much to 7 = like very much
Astringency	Drying, shrinking sensation of oral tissues. Example: alum in water	1 = lowest intensity to 10 = highest intensity	1 = dislike very much to 7 = like very much
Off-taste	Open scale with space allotted for a write-in descriptor to be used when an unanticipated (and abnormal) flavor is observed	Detected or not detected	Not applicable
**OVERALL GRADE**			
Overall grade	Overall quality (Martinez-Villaluenga et al. 2012)	1 = lowest intensity to 10 = highest intensity	1 = dislike very much to 7 = like very much

Supplementary Table S2. Sensory scores of sauerkraut samples from 'Brgujski', 'Žminjski' and commercial sauerkrauts (CS-1, CS-2, and CS-3) obtained by the QDA method.

Sensory attributes	Sauerkraut samples					
	'Brgujski'	'Žminjski'	CS-1	CS-2	CS-3	*p-value*
Color white	0 ± 0 d	0 ± 0 d	4.6 ± 1.8 c	7.9 ± 0.7 a	6.1 ± 1.7 b	***
Color red	4.6 ± 1 a	4.8 ± 1.1 a	0 ± 0 b	0 ± 0 b	0 ± 0 b	***
Fresh cabbage odor	4 ± 1.7 a	3.8 ± 1.7 a	4.1 ± 1.7 a	2.5 ± 0.7 b	3.6 ± 1.7 a	*
Sulfur odor	7.2 ± 0.8	6.9 ± 1.4	6.7 ± 1.7	6.5 ± 1	7 ± 1.3	ns
Off-odor	0.1 ± 0.3	0.3 ± 0.9	0.1 ± 0.3	0.9 ± 1.4	0.3 ± 0.5	ns
Hardness	8.3 ± 0.4 a	8.3 ± 0.9 a	7.2 ± 1 ab	5 ± 1.2 c	6 ± 2.9 bc	***
Crunchiness	8.7 ± 0.7 a	8.7 ± 0.8 a	7.1 ± 1.1 b	5.6 ± 1.4 c	6 ± 2.5 bc	***
Juiciness	8.2 ± 0.7 a	8.4 ± 0.8 a	7.3 ± 1 b	7 ± 0.8 b	7 ± 1 b	***
Fresh cabbage taste	3.9 ± 1.7 a	4.4 ± 1.6 a	4.2 ± 1.7 ab	3.1 ± 0.8 b	3.6 ± 1.9 ab	*
Sulphur taste	7.5 ± 1.7	7.1 ± 1.9	7.5 ± 0.6	6.8 ± 1	6.4 ± 1.2	ns
Sweetness	3.2 ± 1.9	3.8 ± 1.9	2.8 ± 1.8	2.8 ± 1.1	2.8 ± 2	ns
Saltiness	7 ± 2 ab	6.3 ± 2.3 b	6.6 ± 1.3 b	7.9 ± 0.7 a	6.2 ± 2.3 b	*
Sourness	7.8 ± 0.8	7.3 ± 1.5	7.7 ± 1.2	8 ± 0.6	7.2 ± 0.9	ns
Bitterness	2.2 ± 0.9	2.7 ± 1.4	3.3 ± 1.1	2.8 ± 0.9	3.4 ± 2.2	ns
Astringency	2.1 ± 0.8	2.6 ± 1.3	3.2 ± 1.2	2.4 ± 1.1	2.7 ± 1.1	ns
Off-taste	0.1 ± 0.3	0.1 ± 0.3	0.1 ± 0.3	0.3 ± 0.7	0.1 ± 0.3	ns
Overall quality	8.4 ± 0.6 ab	8.5 ± 1.3 a	7.6 ± 0.8 bc	5.9 ± 0.8 d	6.8 ± 0.7 c	***

The means (± standard deviation) with different letters indicate homogenous groups in Tukey's *post-hoc* test

*p≤0.05 , ** p≤0.01, *** p≤0.001, ns: non-significant

Supplementary Table S3. Sensory scores of sauerkraut samples from 'Brgujski', 'Žminjski' and commercial sauerkrauts (CS-1, CS-2, and CS-3) obtained on the Hedonistic scale

Sensory attributes	Sauerkraut samples					*p-value*
	'Brgujski'	'Žminjski'	CS-1	CS-2	CS-3	
Color	5.4 ± 0.8	5.3 ± 1.1	5.9 ± 0.7	5.2 ± 0.8	5.6 ± 1.1	ns
Sulfur odor	5.7 ± 0.7 a	4.9 ± 1.3 ab	5.6 ± 1 a	4.5 ± 0.8 b	4.7 ± 1.2 b	*
Off-odor	0 ± 0	0.1 ± 0.3	0 ± 0	0.6 ± 1.1	0.3 ± 0.9	ns
Hardness	6.2 ± 0.4 a	6.5 ± 0.7 a	5.6 ± 0.9 a	4.5 ± 1.3 b	4.2 ± 1.8 b	***
Crunchiness	6.5 ± 1 a	6.6 ± 0.7 a	5.9 ± 0.9 ab	5.1 ± 1.2 bc	4.6 ± 1.4 c	***
Juiciness	6.5 ± 0.6 a	6.1 ± 0.9 ab	5.7 ± 0.8 bc	5.9 ± 0.3 ab	5.1 ± 1.2 c	**
Fresh cabbage taste	5.3 ± 0.9 a	4.9 ± 1 ab	4.5 ± 0.8 bc	3.9 ± 0.7 c	4.2 ± 0.8 c	***
Sulphur taste	5.8 ± 1.2	5.3 ± 1.2	5.3 ± 0.9	5.1 ± 0.7	5 ± 0.8	ns
Sweetness	5.4 ± 1.1 a	4.9 ± 0.7 ab	4.5 ± 1.4 bc	3.8 ± 1.2 d	4.1 ± 1 cd	***
Saltiness	6.1 ± 1.5 a	5.7 ± 1.7 ab	5 ± 1.4 bc	4.5 ± 1.4 c	5 ± 1.7 bc	*
Sourness	6 ± 1.1	5.9 ± 1.2	5.8 ± 0.6	5.3 ± 1.3	5.2 ± 0.9	ns
Bitterness	4.6 ± 1.6	4.3 ± 1.4	4.4 ± 1.4	3.8 ± 0.8	4.6 ± 0.8	ns
Astringency	4.9 ± 1.1	4.6 ± 1	4.9 ± 1.2	4.3 ± 1.1	4.3 ± 1.1	ns
Off-taste	0 ± 0	0 ± 0	0 ± 0	0.2 ± 0.6	0 ± 0	ns
Overall quality	6.2 ± 0.6 a	6.3 ± 1.3 a	5.4 ± 0.7 b	4.7 ± 0.6 c	5.3 ± 0.4 bc	***

The means (± standard deviation) with different letters indicate homogenous groups in Tukey's *post-hoc* test

*p≤0.05 , ** p≤0.01, *** p≤0.001, ns: non-significant

Supplementary Table S4. ANOVA of phytochemical and physicochemical parameters with PLS-DA VIP scores equal or higher than one. The means (± standard deviation) with different letters indicate homogenous groups in Tukey's *post-hoc* test. *p≤0.05 , ** p≤0.01, *** p≤0.001, ns: non-significant

Parameter (VIP)	Cultivar			Storage conditions			Cultivar*Storage conditions				
							'Žminjski'		'Brgujski'		
	'Žminjski'	'Brgujski'	*p*-value	Ambient storage	Cold storage	*p*-value	Ambient storage	Cold storage	Ambient storage	Cold storage	*p*-value
FRAP (1) (nmol TE/100g FW)	129 ± 15	136 ± 21	ns	118 ± 10	147 ± 10	**	116 ± 1	143 ± 5	121 ± 15	152 ± 13	ns
1-Decanol (2) (normalised peak area)	24886 ± 12141	27675 ± 9949	ns	35072 ± 7366	17489 ± 3361	**	34115 ± 10326	15656 ± 2510	36029 ± 5125	19322 ± 3443	ns
ethyl octanoate (3) (normalised peak area)	52664 ± 20429	32154 ± 9690	**	54695 ± 18751	30123 ± 7057	***	69871 ± 12449	35458 ± 395	39518 ± 5747	24789 ± 6243	ns
DPPH (4)	116 ± 8	113 ± 8	ns	108 ± 4	120 ± 5	**	109 ± 4	122 ± 3	107 ± 5	118 ± 6	ns
2-isothiocyanatobutane (5) (normalised peak area)	37845 ± 29302	16293 ± 4030	***	41630 ± 25258	12508 ± 2587	***	64514 ± 3528 a	11175 ± 527 c	18745 ± 3379 b	13840 ± 3336 bc	***
(L) (6)	57.4 ± 1.4	53.5 ± 3.4	**	57.2 ± 1.6	53.8 ± 3.7	**	57.9 ± 2.1 a	57 ± 0.3 a	56.5 ± 0.7 a	50.6 ± 1.7 b	*
2-undecenal (7) (normalised peak area)	235775 ± 218475	32798 ± 9408	***	233586 ± 220813	34987 ± 10784	***	434118 ± 34235 a	37433 ± 11719 b	33055 ± 9254 b	32541 ± 11638 b	***
(*a*) (8)	10.3 ± 0.7	20.7 ± 3.2	***	14.3 ± 3.8	16.7 ± 7.7	***	10.9 ± 0.3 c	9.7 ± 0.4 d	17.8 ± 0.2 b	23.7 ± 0.5 a	***
ethyl hexadecanoate (9) (normalised peak area)	457689 ± 108710	1920046 ± 261200	***	1110794 ± 640133	1266941 ± 968884	**	531119 ± 114670 c	384260 ± 14824 c	1690469 ± 56617 b	2149623 ± 96135 a	***
(Z)-3-Octen-1-ol acetate (10) (normalised peak area)	285473 ± 208690	138138 ± 56751	***	101834 ± 37920	321777 ± 171272	***	100072 ± 20434 b	470875 ± 73075 a	103597 ± 56285 b	172678 ± 36116 b	***
EC (11) (µS/cm)	119 ± 27	107 ± 4	**	99 ± 7	127 ± 18	***	94 ± 4 c	143 ± 4 a	105 ± 4 b	110 ± 3 b	***
